# Gut microbiome disruption in Tanzanian pulmonary tuberculosis patients: links to treatment, nutritional status, and host immunity

**DOI:** 10.1186/s12866-026-04882-3

**Published:** 2026-03-26

**Authors:** Vesla I. Kullaya, Sylvia Rofael, Scott K. Heysell, Tania A. Thomas, Nehemiah Ongeso, Kimita Gathii, Bibie Said, Stellah Mpagama, Blandina T. Mmbaga, Timothy D. McHugh

**Affiliations:** 1 Kilimanjaro Clinical Research Institute (KCRI), Kilimanjaro Christian Medical Centre (KCMC), Moshi, Tanzania; 2 Department of Medical Biochemistry and Molecular Biology, KCMC University, Moshi, Tanzania; 3 Division of Infection and Immunity, Centre for Clinical Microbiology, University College London, London, UK; 4 Faculty of Pharmacy, Alexandria University, Alexandria, Egypt; 5 Division of Infectious Diseases & International Health, Department of Medicine, University of Virginia, Charlottesville, VA USA; 6 Kenya Medical Research Institute/Walter Reed Army Institute of Research-Africa/Basic Science Laboratory, Kisumu, Kenya; 7 Kibong’oto Infectious Diseases Hospital, Mae, Sanya Juu, Siha, Tanzania; 8 School of Medicine, Institute of Public Health, KCMC University, Moshi, Tanzania

**Keywords:** Tuberculosis, Gut microbiome, Anti-tuberculosis treatment, Inflammatory cytokines, Malnutrition, Microbiome dysbiosis

## Abstract

Tuberculosis (TB) and its treatment disrupt the gut microbiome, potentially impacting immune regulation and treatment outcomes. However, host-microbial interactions during TB disease are not well characterized. This cross-sectional study investigated the gut microbiome of pulmonary tuberculosis patients—both newly diagnosed and those treated for two months—compared to healthy controls in Tanzania. TB treatment was associated with reduced microbial diversity, distinct clustering, and depletion of *Euryarchaeota* and *Cyanobacteria*. Treated patients also showed increased *Fusobacteria* and *Tenericutes* compared to controls. In contrast, newly diagnosed patients had gut microbiomes similar to controls. Malnutrition and elevated inflammatory cytokines were more common in newly diagnosed patients. Within this group, gut microbial composition varied by nutritional status, cytokine levels, and exposure to pre-diagnosis non-antituberculous antibiotics. Our findings demonstrate that TB treatment significantly alters the gut microbiome and suggest complex interactions between gut microbial populations, host inflammation, nutrition, and treatment history. This study also underscores the need for more microbiome research in Sub-Saharan Africa to better understand immune-microbiome dynamics in TB.

**Supplementary Information:**

The online version contains supplementary material available at 10.1186/s12866-026-04882-3.

## Introduction

Tuberculosis (TB) remains a major global health challenge and is the leading infectious cause of death. Low- and middle-income countries (LMICs) where comorbidities like HIV and malnutrition are prevalent, account for 99% of newly diagnosed TB cases each year [1]. Undernutrition promotes progression from tuberculosis infection to symptomatic disease worldwide. Emerging evidence suggests that gut microbiota homeostatic disruption and environmental enteropathy may act as biological contributors to undernutrition-related TB disease [2–4]. Furthermore, TB therapy contributes to gut microbiome dysbiosis, potentially exacerbating post-TB non-communicable diseases (NCDs) through microbiome-mediated pathways [5–7]. This is particularly concerning in Sub Saharan Africa, where TB-HIV co-infection rates exceed 30% in some regions, and both diseases independently alter gut microbial ecology.

The gut microbiome plays a crucial role in modulating inflammatory and metabolic processes through microbial metabolites such as short-chain fatty acids (SCFAs), lipopolysaccharides (LPS), and endotoxins. These bioactive molecules can cross the intestinal epithelial barrier and interact with host cells, potentially contributing to inflammatory and metabolic disorders, including diabetes and cardiovascular diseases [5, 8–10].

Microbiome dysbiosis has been linked to pro-inflammatory states that exacerbate TB disease severity, as well as anti-inflammatory effects that could modulate disease progression [11–17]. Additionally, Wipperman et al.. reported that anti-TB therapy selectively affects the abundance of immunoregulatory microbes rather than overall microbial diversity, with dysbiosis persisting for up to 1.2 years post-treatment [18].

Despite Sub-Saharan Africa shouldering a significant TB burden, few studies have examined the effects of TB infection and anti-TB therapy on the gut microbiome and its downstream impact on the immune system in this population. Existing research highlights substantial gaps in understanding the mechanistic role of gut microbiota in TB pathophysiology across the disease continuum—from active disease to treatment and recovery. Consequently, key microbial biosignatures remain unintegrated into population-specific TB prevention and management strategies such as intensified nutritional support.

The present study aimed to investigate the interaction between the gut microbiome and inflammatory responses in pulmonary TB infection by characterizing the effects of TB and anti-TB therapy on gut microbiome composition and diversity, as well as their associations with inflammatory cytokines in Tanzanian patients with pulmonary TB in contrast with healthy controls. Understanding these interactions will provide insights into microbiota-mediated immune regulation and inflammatory responses, potentially identifying microbial and inflammatory biomarkers that could serve as targets for adjunctive therapies and/or enhance nutritional interventions.

## Results

### Demographic and clinical characteristics of study participants

A total of 50 participants aged between 30 and 44 years old were recruited. Characteristics of study participants are summarized in Table 1. Newly diagnosed TB patients showed significant differences in key health indicators compared to controls. The cohort exhibited marked age disparity, with TB patients being older (median [IQR]) 43.0 [40.0–44.7] years) than controls (30.0 [28.0–36.2] years; *p* = 0.0015). Anthropometric measures revealed lower BMI values in both newly diagnosed patients (19.4 [17.4–21.6]) and those on treatment (20.2 [19.4–24.1]) compared to controls (24.7 [20.7–25.7]; *p* = 0.0038 and *p* = 0.049, respectively). Clinical assessment showed 40% of newly diagnosed patients were malnourished (versus 15% in the on-treatment group), and 35% were smokers. Notably, 60% of newly diagnosed patients reported pre-diagnosis antibiotic use (versus 15% in the on-treatment group; *p* = 0.003), predominantly amoxicillin.

**Table 1:** Demographic, anthropometric, and clinical characteristics of TB patients and controls

	Controls *n* = 10	Newly diagnosed patients *n* = 20	Patients on treatment *n* = 20	*P*-value
Age in years (median, IQR)	30.0 (28.0 – 36.2)	43.0 (40.0 – 44.7)	40.5 (28.2 – 44.0)	0.018ª
Males (N, %)	6 (60%)	19 (95%)	18 (90%)	0.015*
Body Mass Index (median, IQR)	24.7 (20.7–25.7)	19.4 (17.4–21.6)	20.2 (19.4–24.1)	0.013ª
Malnourished, BMI < 18.5 (N, %)	0	8 (40%)	3 (15%)	0.020*
Smoker (n, %)	0	7 (35%)	5 (25%)	0.033*
Used empirical antibiotics before TB diagnosis (n, %)	*NA*	12 (60%)	3 (15%)	0.003^*x*^
Amoxicillin	*NA*	7 (35%)	3 (15%)	
Other antibiotics	*NA*	5 (25%)	0	
Systolic blood pressure, (median, IQR) mmHg	*NA*	124.0 (110.0–127.0)	129.0 (117.0–135.7)	
Diastolic blood pressure, (median, IQR) mmHg	*NA*	76.5 (70.2–84.0)	78.5 (75.2–83.5)	
Plasma Cytokines (median, IQR) pg/ml				
IFNγ	8.20 (2.87–8.20)	17.60 (8.20–34.68)	8.20 (2.35–8.20)	0.0018^a^
TNFα	3.65 (2.95–5.12)	8.70 (6.05–13.28)	5.20 (3.57–7.72)	0.0012^a^
IL-1β	0.25 (0.17–0.75)	4.15 (1.65–9.07)	0.50 (0.15–0.90)	< 0.0001^a^
IL-6	0.40 (0.17–0.70)	13.60 (6.55–24.73)	1.60 (1.00–4.05)	< 0.0001^a^
IL-10	0.40 (0.35–0.97)	0.80 (0.42–1.00)	0.70 (0.50–1.52)	
Cytokine profile (high vs. low)				
High_IFNγ (> 17.60) pg/ml	0	10 (50%)	0	
High_TNFα (> 8.70) pg/ml	0	10 (50%)	0	
High_IL-1β (> 4.15) pg/ml	0	10 (50%)	0	
High_IL-6 (> 13.60) pg/ml	0	10 (50%)	0	

*NA*  not applicable

ª*p*-value by Kruskal-Wallis test

^*^p-value by Chi square (controls vs. patients); ^x^*p*-value by Chi square (newly diagnosed patients vs. patients on treatment)

### Group differences in systemic cytokine levels and microbial load in TB infection

**Fig. 1:**
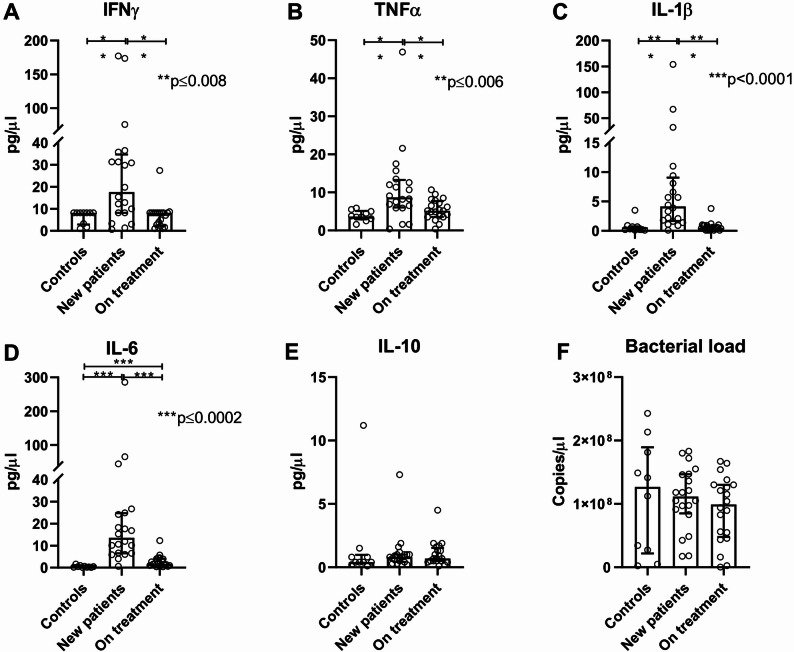
Plasma cytokines and bacterial density. Scatter dot plots showing median and interquartile range of levels of plasma cytokines (**A**-**E**) and quantity of bacteria in stool samples (**F**). Differences between groups were calculated using Mann-Whitney test

Cytokines measured from plasma samples revealed distinct profiles across groups. Pro-inflammatory cytokines - IFNγ, TNFα, IL-1β and IL-6 - were significantly higher in newly diagnosed tuberculosis patients compared to both controls and patients on treatment (Fig. 1A-D). While most cytokine levels normalized with treatment, IL-6 remained elevated in the on-treatment group (Fig. 1D), suggesting persistent activation of IL-6-associated inflammatory pathways, NF-ĸβ or JAK-STAT3. IL-6 correlated positively with IL-1β and TNFα (*r* = 0.61 *p* = 0.012, FDR corrected, for both comparisons) but not with IFNγ, which is regulated through distinct immune pathways and cellular sources. Levels of anti-inflammatory cytokine IL-10 were very low across all groups and without significant differences (Fig. 1E). The total number of copies of 16 S rRNA gene per microlitre of DNA extract was used as a proxy for the total bacterial density in the stool samples. There was a trend in the decline of bacterial density in new TB patients towards patients exposed to anti-TB medication as compared to healthy controls although these differences were not statistically significant (Fig. 1F).

### Microbiome diversity and composition shifts in TB progression and treatment

We processed a total of 50 stool samples of which 49 were successfully sequenced for the 16 S rRNA (V3-V4) marker with the following distribution across subject groups; controls (*n* = 10), newly diagnosed patients (*n* = 20) and patients on treatment (*n* = 19) yielding 12,059,450 raw reads. The median sequencing depth was 147,561 reads per sample and a minimum of 61,947 reads per sample. After preprocessing steps—including quality control, denoising, and chimera removal—a total of 8,467,850 quality reads were retained (Supplementary Table S1), resulting in 4,275 Amplicon Sequence Variants (ASVs), classified into 11 phyla, 15 classes, 46 families, 128 genera, and 216 species after removal of unassigned values. The relative abundance of each phylum is shown in Supplementary Fig. 1.

**Fig. 2:**
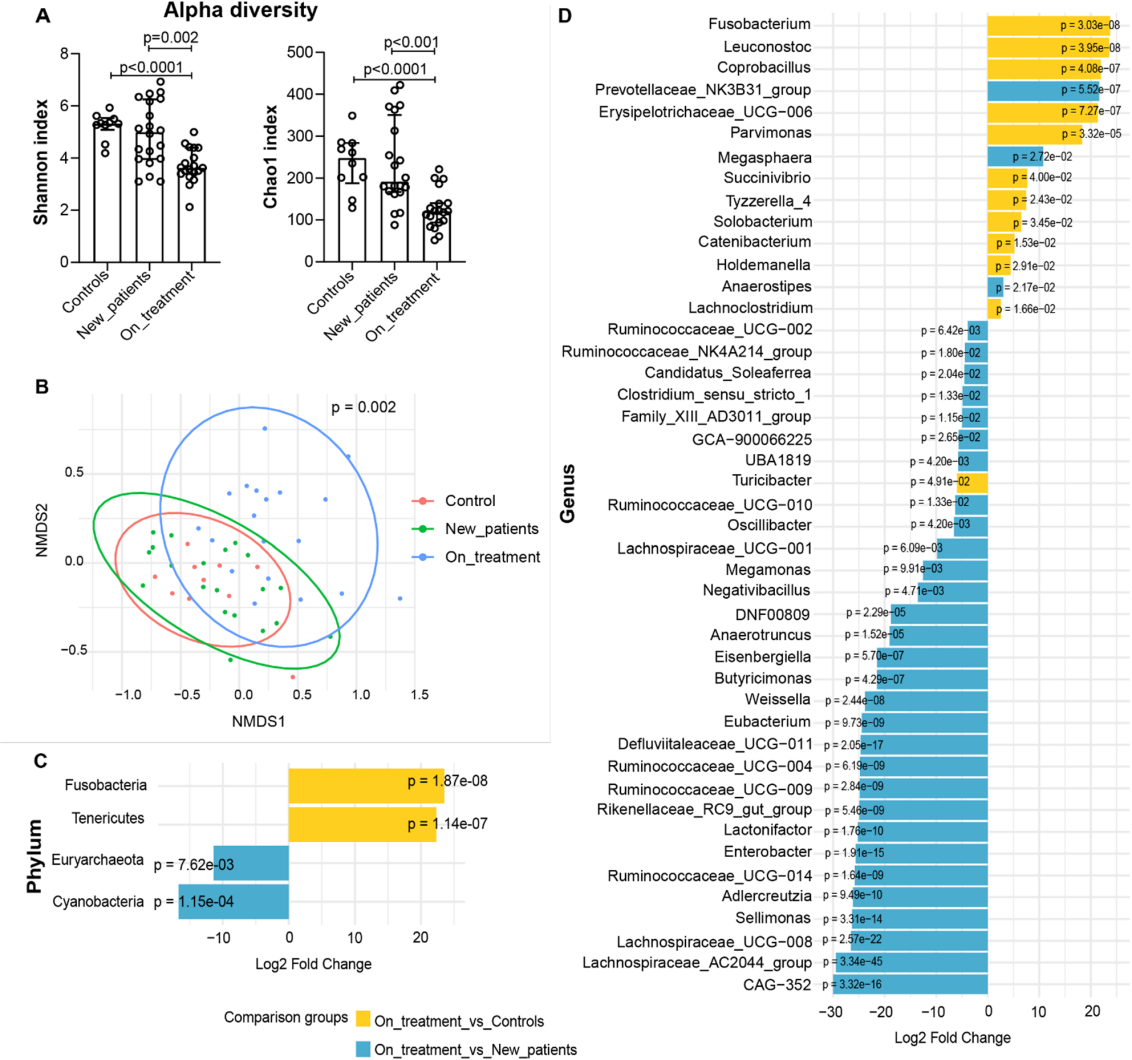
Gut microbial diversity between patient groups and controls. **A** Alpha diversity. Scatter dot plots show median and interquartile range of Shannon and Chao-1 indices. Differences between groups were calculated using Mann-Whitney test. **B** Beta diversity: Non-metric Multidimensional Scaling (NMDS) plot showing differences in microbial communities between samples. Dots represent individual samples, and the distance between points reflects the dissimilarity in microbial community composition based on Bray-Curtis distances. Ellipses indicate 95% confidence intervals around the group centroids, calculated using a multivariate t-distribution, to represent the variation in community composition within each group. The NMDS had a stress value of < 0.17, indicating a fair representation of the data in two dimensions. *P*-value indicates a pairwise significant difference between newly diagnosed patients (New_patients) and patients on treatment (R^2^ = 0.0475; *p* = 0.002,) adjusted for age, sex and BMI. **C**-**D** DESeq2 bar plots showing differentially abundant phyla (**C**) and genera (**D**) between groups. The x-axis represents the log₂ fold change in abundance, and the y-axis lists taxa with statistically significant differences (FDR-adjusted *p*-value < 0.05). Positive log₂ fold change values indicate taxa enriched in patients on treatment, while negative values indicate taxa depleted in this group. Bars are coloured by comparison group: yellow for patients on treatment vs. controls, and blue for patients on treatment vs. newly diagnosed patients

Patients undergoing TB treatment showed significantly reduced alpha diversity compared with both controls and newly diagnosed patients (Fig. 2A). Shannon diversity was lower in treated patients (median 3.62, IQR 3.40–4.39) than in controls (5.32, IQR 5.08–5.53; *p* < 0.0001) and newly diagnosed patients (5.00, IQR 3.97–6.25; *p* = 0.002). Similarly, Chao1 richness was reduced in treated patients (121.0, IQR 94.0–140.0) compared with controls (248.0, IQR 187.5–284.0; *p* < 0.0001) and newly diagnosed patients (191.5, IQR 166.8–350.6; *p* < 0.0001). Although alpha diversity indices were lower in newly diagnosed patients compared with controls, these differences did not reach statistical significance.

Beta diversity analysis revealed a significant difference in microbial community composition between groups (pseudo-F = 1.49, R^2^ = 0.061; *p* = 0.002), also after correcting for age, sex and BMI (R^2^ = 0.12; *p* = 0.03), indicating that group differences explain approximately 6.1% of the total variation in microbial community structure. Pairwise comparison further showed a significant difference between newly diagnosed patients and patients on treatment (R^2^ = 0.0475; *p* = 0.002, adjusted for age, sex, and BMI) indicating distinct or distant microbial communities between the two groups (Fig. 2B). Although adjustment for age, sex, and BMI reduced potential confounding, residual confounding cannot be fully excluded given the cross-sectional design and modest sample size.

DESeq2 analysis revealed that diversity differences were primarily driven by higher abundance of phyla *Fusobacteria* and *Tenericutes* in patients on treatment as compared to controls (*p* < 1 × 10^− 6^), and depletion of phyla *Euryarchaeota* and *Cyanobacteria* in patients on treatment as compared to newly diagnosed patients (*p* < 0.01) (Fig. 2C). At genus level, patients on treatment had higher abundance of 11 genera led by *Fusobacterium*, *Leuconostoc* and *Coprobacillus* as compared to controls, and 3 genera including *Prevotellaceae_NK3B31_group*,* Megasphaera* and *Anaerostipes* as compared to newly diagnosed patients. Furthermore, patients on treatment had lower abundance of genera *Turicibacter* as compared to controls, and lower abundance of 30 genera led by *Lachnospiraceae_AC2044_group*, *Lachnospiraceae_UCG-008*,* Defluviitaleaceae_UCG-011*,* CAG-352* and *Enterobacter* as compared to newly diagnosed patients (Fig. 2D). A list of significantly differentially abundant taxa is provided in Supplementary Table 2.

### Characteristics of the gut microbiome in TB patients before treatment

Finally, we assessed the characteristics of the gut microbial community in newly diagnosed TB patients prior to treatment initiation in order to understand how the microbial composition relates to environmental, clinical and inflammatory markers. Gut microbiome profiles were examined in newly diagnosed patients stratified by nutritional status, inflammatory cytokine levels, and exposure to pre-diagnosis empirical non-antituberculous antibiotics, which was treated as a key contextual modifier rather than an exclusion criterion. Alpha diversity analysis showed no statistically significant differences between these groups however we observed a tendency of higher variation in microbial diversity in groups of patients with malnutrition, patients exposed to pre-diagnosis antibiotics, patients with low TNFα levels and patients with low IL-6 levels as compared to their counterparts (Fig. S2). Beta diversity analysis revealed a marginal difference in microbial community composition between malnourished and nourished patients (pseudo-F = 1.23, R^2^ = 0.1877; *p* = 0.05, and between patients with high vs. low IL-6 levels (*p* = 0.06) corrected for age and BMI. Observed trends in the findings indicate that these factors may have pathophysiological links to the gut microbiome. However, a larger and more prospective cohort is needed to evaluate individual patient-level changes over time.

**Fig. 3:**
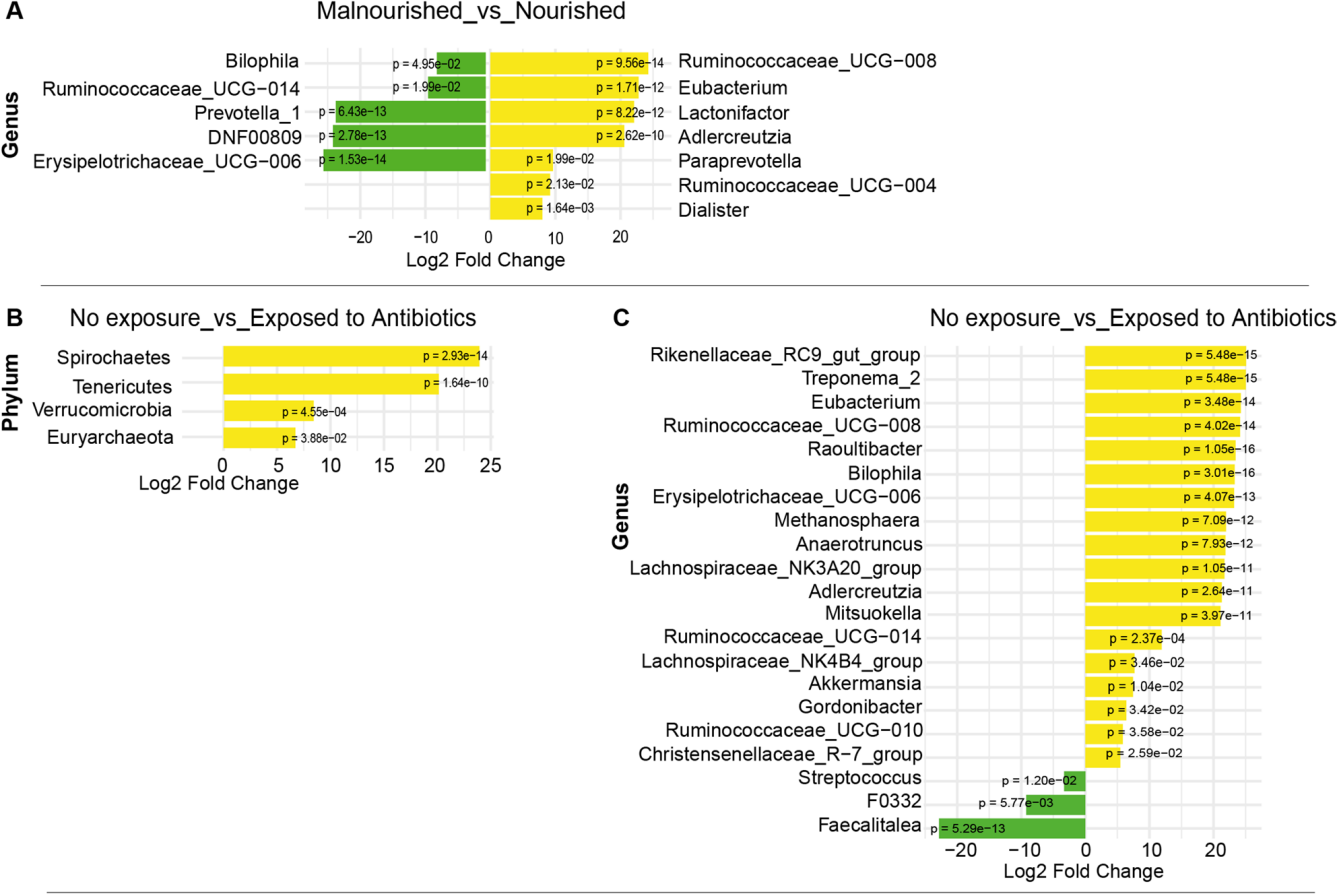
Differentially abundant taxa across newly diagnosed TB patients with different nutritional status and medication history. DESeq2 bar plots showing differentially abundant taxa across newly diagnosed TB patients with and without malnutrition (**A**), with and without exposure to pre-diagnosis non-antituberculous antibiotics (**B**-**C**). The x-axis represents the log₂ fold change in abundance, and the y-axis lists taxa (phylum or genus) with statistically significant differences (FDR-adjusted *p*-value < 0.05). Yellow colour indicates taxa enrichment, while green colour indicates taxa depletion in the comparison group

Despite the absence of robust differences in the overall alpha and beta diversities, DESeq2 analysis showed higher abundance of 7 genera in nourished newly diagnosed patients led by *Ruminococcaceae_UCG-008*,* Eubacterium* and *Lactonifactor* (*p* < 1e-11) and depletion of 5 genera led by *Erysipelotrichaceae_UCG-006*,* DNF00809* and *Prevotella_1* (*p* < 1e-12) (Fig. 3A). No differences in the abundance of phyla were observed. Patients exposed to pre-diagnosis non-antituberculous antibiotics had enrichment of 4 phyla including *Spirochaetes* and *Tenericutes* (*p* < 1e-09), and *Verrucomicrobia* (*p* < 0.001) and *Euryarchaeota* (*p* = 0.04) (Fig. 3B). At genus level, patients exposed to pre-diagnosis antibiotics had enrichment of 18 genera led by *Raoultibacter*,* Bilophila*,* Rikenellaceae_RC9_gut_group*,* Treponema_2*, and *Eubacterium* (*p* < 1e-13); and depletion of 3 genera including *Faecalitalea* (*p* < 1e-12), *F0332* (*p* < 0.01) and *Streptococcus* (*p* = 0.012) (Fig. 3C).

**Fig. 4:**
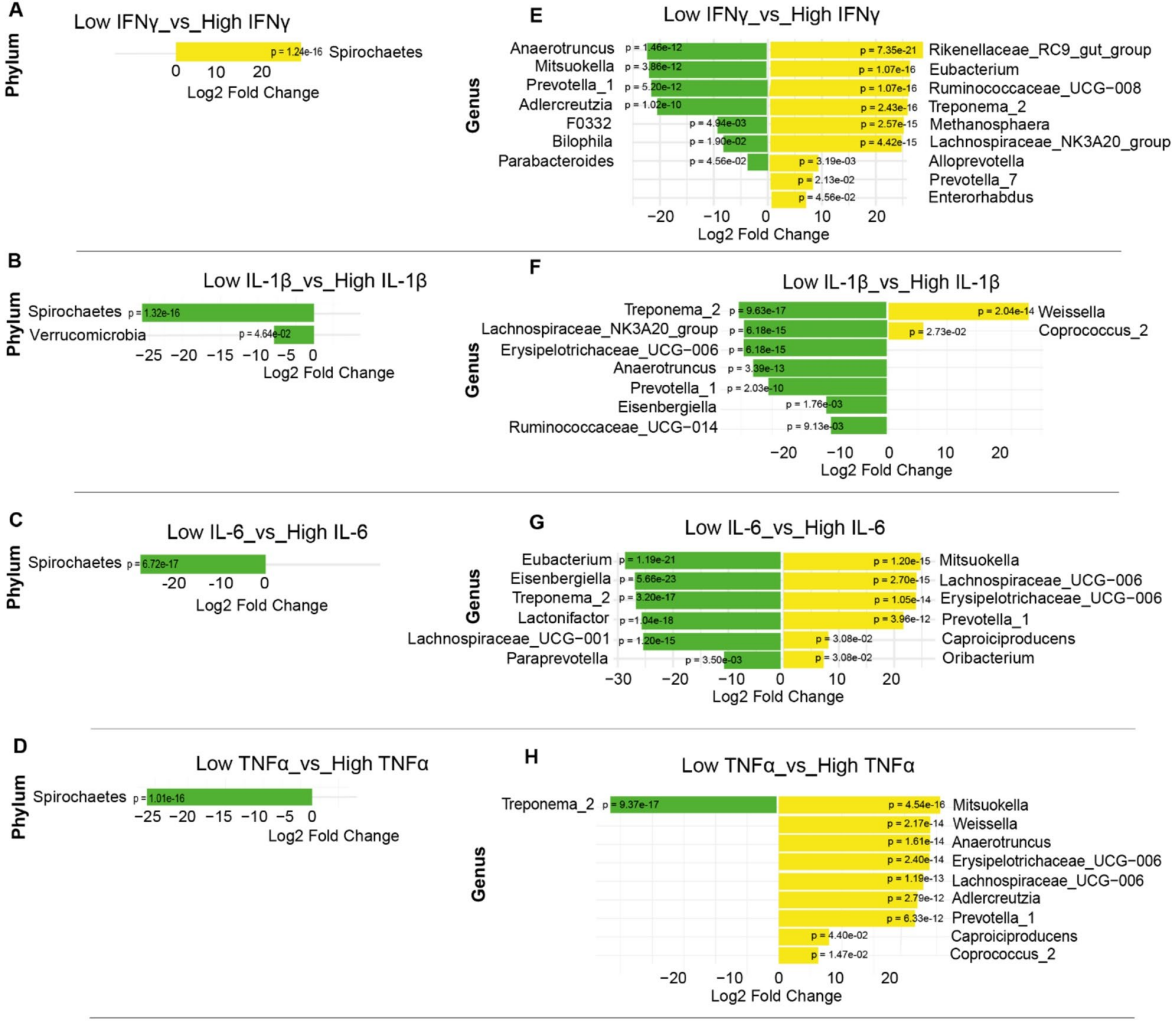
Differentially abundant taxa across newly diagnosed TB patients with different cytokine levels. DESeq2 bar plots showing differentially abundant taxa across newly diagnosed TB patients with low and high IFNγ levels (**A**, **B**); with low and high IL-1β levels (**C**, **D**), with low and high IL-6 levels (**E**, **F**) and with low and high TNFα levels (**G**, **H**). The x-axis represents the log₂ fold change in abundance, and the y-axis lists taxa (phylum or genus) with statistically significant differences (FDR-adjusted *p*-value < 0.05). Yellow colour indicates taxa enrichment, while green colour indicates taxa depletion in the comparison group

With regard to inflammatory cytokines, we observed cytokine-specific differences in microbial abundance, particularly between IFNγ and other cytokines, most notably IL-6 and TNFα. Specifically, the phylum *Spirochaetes* was significantly enriched in newly diagnosed patients with high IFNγ levels (*p* < 1.24e-16), as well as in those with low levels of IL-6, IL-1β, and TNFα (*p* < 1e-15) (Fig. 4A–D). At the genus level, patients with high IFNγ levels showed enrichment of *Treponema_2*, which was also enriched in patients with low IL-6, IL-1β, and TNFα levels; *Eubacterium*, enriched in those with low IL-6; and *Lachnospiraceae_NK3A20_group*, enriched in patients with low IL-1β (Fig. 4E–H).

In contrast, genera such as *Mitsuokella* and *Prevotella_1*, which were depleted in patients with high IFNγ, were enriched in those with high IL-6 and TNFα levels. *Anaerotruncus* and *Adlercreutzia* were also enriched in patients with high TNFα. Some overlaps were observed, including the depletion of *Prevotella_1* and *Anaerotruncus* in patients with high levels of both IFNγ and IL-1β. Additional differences associated with IL-1β were noted: for example, *Erysipelotrichaceae_UCG-006* and *Prevotella_1*, which were depleted in patients with high IL-1β, were enriched in those with high IL-6 and TNFα, and *Anaerotruncus* was again enriched in those with high TNFα (Fig. 4F–H).

## Discussion

In this cross-sectional study we assessed the genetic diversity and composition of the gut microbiome in adult Tanzanian patients newly diagnosed with TB before initiation of treatment in comparison to healthy controls and a group of TB patients who had been exposed to the intensive phase of anti-TB therapy for two months. Our findings showed that the structure of the gut microbiome of TB patients exposed to anti-TB medication differs from that of untreated patients and from healthy controls with significantly reduced diversity and significant shifts in microbial community structure across groups; however, the cross-sectional design precludes direct inference on within-individual microbiome changes over time. Importantly, we observed differential enrichment or depletion of specific microbial taxa in those with and without TB disease and those with and without exposure to TB treatment. Furthermore, we identified associations between gut microbiome profiles and host clinical features, including nutritional status, pre-diagnosis non-antituberculous antibiotic exposure, and systemic inflammatory cytokines.

Our findings suggest a profound alteration of the gut microbiome during TB treatment which may have been driven by the prolonged or intensive use of antibiotics of four different antibiotic classes with distinct mechanisms of antimicrobial action. These findings contribute to the sparse evidence that the anti-tuberculous drug may have broader impact on other bacterial genera distinct from mycobacteria. Rifampicin, and the class of rifamycins, are more widely recognized as a broader spectrum antibiotic in the regimen that inhibits bacterial RNA polymerase of many bacterial species and may affect the composition and resilience of the gut microbial community [19], yet our findings of significant alteration in both microbial diversity and community structure after exposure to TB drugs suggests the combination regimen may have a broader impact than previously appreciated. In contrast, previous studies have demonstrated that combination TB medication has a prolonged effect on the microbial community structure yet no or only marginal effect on the microbial diversity [18–20]. Studies in mice have shown that monotherapy with rifampicin reduces microbial diversity while monotherapy with isoniazid and pyrazinamide cause alteration in taxa abundance [19]. Our findings indicate that the impact of TB medication on the gut microbiome results from the combined effect of drugs in the TB regimen, influencing both microbial diversity and composition. The absence of significant differences in total bacterial load alongside marked reductions in alpha diversity suggests that TB treatment primarily drives compositional restructuring of the gut microbiome rather than depletion of total bacterial biomass.

The observed enrichment of phyla such as Fusobacteria and Tenericutes in patients on treatment, alongside the depletion of Euryarchaeota and Cyanobacteria, further indicates a treatment related dysbiosis. At the genus level, the overrepresentation of Fusobacterium and Leuconostoc and the underrepresentation of most genera belonging to phylum Firmicutes may reflect a loss of beneficial short-chain fatty acid producers and an increase in pro-inflammatory or opportunistic taxa. These shifts have been associated with compromised gut barrier function and increased systemic inflammation [21, 22], as suggested by our cytokine analyses.

In newly diagnosed patients, we observed microbial compositional shifts, particularly in malnourished individuals and those with prior use of empirical antibiotics emphasizing on the role of diet and antimicrobial pressure in shaping gut ecology. Amoxicillin, a relatively broad-spectrum antibiotic targeting numerous gut bacterial genera, was the most commonly used antibiotic prior to TB diagnosis. Amoxicillin is frequently prescribed for pneumonia, where the patient’s response to amoxicillin often guides clinical suspicion for tuberculosis. Several other commonly used antibiotics in the Tanzanian setting have been reported to alter the gut microbiome [23]. This observation suggests that while richness and evenness may remain relatively stable, the microbial community structure is sensitive to host nutritional and treatment history.

The gut microbiome plays a key role in host immunity and inflammation, primarily through the ability of certain microbial species to produce enzymes that ferment nutrients into absorbable compounds [24, 25]. This includes the conversion of carbohydrates into short-chain fatty acids (SCFAs) like propionate, butyrate, acetate, and lactate, which exhibit anti-inflammatory and immunomodulatory properties. SCFAs exert their effects by interacting with G protein-coupled receptors, triggering immune responses through signal transduction pathways [10, 26]. Because 16 S rRNA gene sequencing does not permit direct functional characterization, interpretations regarding microbial metabolic or immunoregulatory functions are based on taxonomic inference and existing literature rather than direct measurement. Here, we considered cytokines that play key roles in the pathophysiology of tuberculosis. IFNγ activates macrophages and enhances reactive nitrogen species–mediated intracellular killing of *Mycobacterium tuberculosis*. TNFα is essential for maintaining granuloma integrity and containing the bacteria, but it also contributes to fever and tissue damage. IL-1β promotes inflammation and helps control *M. tuberculosis* replication, while IL-10, an anti-inflammatory cytokine, suppresses excessive immune responses and protects against tissue damage [27–29]. A notable finding was that microbial taxa enriched in patients with high IFNγ levels were depleted in those with elevated IL-6, IL-1β, and TNFα, indicating distinct associations between gut microbiota and specific immune pathways. IFNγ is linked to Th1-type responses via IL-12/JAK-STAT4 and TCR signaling, while IL-6, IL-1β, and TNFα are involved in pro-inflammatory pathways [30–32]. This suggests potential cytokine-specific associations between gut microbial taxa and immune pathways, although unmeasured immune mediators (e.g., IL-17, IL-22) and host genetic factors may also contribute. Although data on Spirochaetes remain limited, their presence in inflammatory conditions associated with gut dysbiosis [33], points to a potential role in mucosal immunity. Despite the absence of direct gut permeability markers such as plasma citrulline or alpha-1 antitrypsin our findings support a biologically plausible link between gut microbiota composition and systemic immunity rather than providing direct evidence of altered gut permeability.

This study is strengthened by its integrative approach, combining gut microbiome analysis with clinical and immunological data. However, it was designed as a pilot, exploratory investigation intended to generate preliminary hypotheses rather than provide definitive inference. Accordingly, subgroup analyses were interpreted cautiously, and the absence of statistical significance for some observed trends likely reflects limited statistical power rather than absence of biological effect. Limitations include lack of longitudinal data, small sample size, and use of 16 S rRNA sequencing, which limited functional interpretation. Subgroup analyses involved small sample sizes, increasing the risk of both false-positive and false-negative findings despite FDR correction; therefore, these results should be interpreted as hypothesis-generating. The cross-sectional design constrained causal inference, precluded analysis of microbiome changes over the course of treatment, and limited linkage with treatment outcomes and environmental factors such as diet. Future studies should adopt longitudinal designs and incorporate functional metagenomic or metabolomic profiling to better link microbiome dynamics with immune modulation and clinical treatment outcomes. Intervention studies exploring the microbiome change over time with intensive micro-macronutrient interventions, and the impact of prebiotics or probiotics as adjuncts to TB therapy are also warranted.

## Conclusion

We report the structure of the gut microbiome in tuberculosis patients in the Tanzanian population. Our study demonstrates that TB and its treatment are associated with marked shifts in gut microbiota diversity and composition, which in turn are associated with patterns of host immune response and clinical factors such as malnutrition and prior exposure to empirical non-antituberculous antibiotics. These findings call for further investigation into the role of gut microbiome in supporting immune homeostasis during TB disease.

## Methods

### Study population and design

We performed a cross-sectional study at Kibong’oto Infectious Diseases Hospital (KIDH) in Kilimanjaro region in Northern Tanzania between July and September 2023. KIDH is a national referral hospital for TB and a centre of excellence for multi-drug resistant (MDR)-TB in the country, caring for approximately 1,000 people with TB/MDR-TB per year [34].

Participants included: (i) newly diagnosed adult patients (≥ 18 years) with sputum XpertMTB/RIF- confirmed *Mycobacterium tuberculosis* infection who were recruited on the same day as TB diagnosis and had not initiated anti-tuberculosis treatment at the time of enrollment; (ii) TB patients who had been exposed to standard anti-TB therapy for 2 months, and (iii) healthy controls. Participants were recruited in a 2:2:1 ratio. Newly diagnosed patients were considered anti-TB treatment-naïve; however, some had received empirical, non-TB antibiotics for respiratory symptoms prior to TB diagnosis. Healthy controls were recruited from hospital staff and patients’ companions. Eligibility was determined through a standardized medical interview assessing current symptoms, medical history, recent illnesses, and medication use. Individuals were considered eligible as healthy controls if they were asymptomatic at the time of recruitment, not taking any medications, and had not used antibiotics within the preceding three months. Controls were not microbiologically screened for tuberculosis. Patients on treatment received a combination of weight-based daily rifampicin, isoniazid, pyrazinamide and ethambutol for the first 2 months, followed by rifampicin and isoniazid for 4 months. Critical illness and HIV co-infection were exclusion criteria. Given that this was a pilot study, a convenience sampling approach was used to determine the sample size.

### Cytokine quantification

Blood samples were collected from all participants using EDTA vacutainer tubes (Cat #: 367525, Becton Dickson, UK) and centrifuged at 3800 rpm for 8 min at room temperature to obtain plasma for cytokine analysis. Plasma samples were stored at -80℃ until day of analysis. Inflammatory cytokines including interferon gamma (IFNγ), tumour necrosis factor alpha (TNFα), interleukin 1 beta (IL-1β) and IL-10 were quantified from plasma without stimulation using a Human High Sensitivity Cytokine A Premixed Magnetic Luminex^®^ Performance Assay (Cat #: FCSTM09, R&D Systems, UK) according to manufacturer’s instructions. For each cytokine, participants with cytokine levels above the median were categorized as High_IFNγ, High_TNFα, High_IL-6, and High_IL-1β, while those with values below the median were categorized as Low_IFNγ, Low_TNFα, Low_IL-6, and Low_IL-1β.

Cytokine concentrations were primarily analyzed as continuous variables and summarized using medians and interquartile ranges. Median-based categorization into high and low groups was applied as an exploratory approach for stratified analyses, given the pilot nature of the study and the lack of established clinical thresholds.

### Stool collection and DNA extraction

Stool samples were collected in sterile containers on day of recruitment, aliquoted and stored at -80℃ until day of DNA extraction. DNA was extracted using the ZymoBIOMICS DNA Miniprep Kit (D4300S, Zymo Research, USA) according to manufacturer’s instructions. Briefly, 200 mg of stool sample was mixed 750 µl lysis solution and mixed in a MP Fastprep bead beater (MP Biomedicals™, Fisher Scientific, UK) at 4.0 m/s for 5 min. Supernatant was treated with DNA binding buffer and filtered through a Zymo-Spin column. DNA was eluted into100 µl DNase/RNase free water. NanoDrop 2000 spectrophotometer (Thermo Scientific, UK) was used to quality control the extracted DNA. The ZymoBIOMICS Microbial DNA Standard (D6305-A, Zymo Research, USA) was used as the extraction control.

### Determination of bacterial density by quantitative PCR

The total bacterial density indicated by the number of copies of 16 S rRNA gene per µl in the stool samples DNA extract was determined by Taq-Man probe assay quantitative PCR (qPCR) using the universal bacterial primers Bact340F and Bact806R which targets the 466 bp fragment (V3-V4 hypervariable regions) of the gene. The master mix was prepared using Platinum^®^ quantitative PCR Supermix-UDG (Thermo-Fisher Scientific, UK). PCR inhibition was tested by inclusion of an internal amplification control (IAC), SPUD A, consisting of 101 bp of the PhyB gene of *Solanum tuberosum*. Reactions were performed on a Qiagen Rotor-gene 6000 machine (Corbett Research UK, Cambridgeshire, UK) using the following protocol; 95 °C for 3 min followed by 40 cycles of 95 °C for 10 s and 60°C for 45 sec. Final concentration was determined by taking the geometric mean of triplicate readings for each sample. The limitation of using this approach is that different bacterial species have varying numbers of 16 S rRNA gene copies per genome. Additionally, the copy number may increase with heightened metabolic activity, leading to potential overestimation of actively growing bacteria. Primers and probes used in the assay are shown on the Table 2.

**Table 2:** Primers and probes for 16S rRNA target and internal amplification control used in qPCR assay

Reagent	5’–3’ Sequence
q16S rRNA Forward Primer (Bact340F)	TCCTACGGGAGGCAGCAGT
q16S rRNA Reverse Primer (Bact806R)	GGACTACCAGGGTATCTAATCTT
q16S rRNA probe	[ROX] CGTATTACCGCGGCTGCTGGCAC [BHQ2]
Spud Forward primer	AACTTGGCTTTAATGGACCTCCA
Spud Reverse primer	ACATTCATCCTTACATGGCACCA
Spud Probe	Cy5-TGCACAAGCTATGGAACACCACGT-BBQ
SpudA	AACTTGGCTTTAATGGACCTCCAATTTTGAGTGTG CACAAGCTATGGAACACCACGTAAGACATAAAAC GGCCACATATGGTGCCATGTAAGGATGAATGT

### 16S rRNA gene sequencing

A sequence library was prepared by first amplifying the V3-V4 regions of the bacterial 16 S rRNA gene by conventional PCR. Each sample was assigned a unique combination of standard Illumina^®^ dual-indexed primers, with P5 and P7 adaptors attached on the forward primer and on the reverse primer respectively (Table 2). The PCR master mix consisted of 0.2 µM of 341 forward primer (CCTACGGGNGGCWGCAG), 0.2 µM of 805 reverse primer (GACTACHVGGGTATCTAATCC), 12.5 µL of KAPA HiFi HotStart ReadyMix containing enzyme and 1.5 mM MgCl_2_ and 5ng DNA template. The following thermo-cycling protocol was used; 95 °C for 5 min, followed by 25 cycles of 95°C for 30 s, 55°C for 40 s and 72°C for 1 min, and a final extension phase at 72°C for 10 min.

The obtained amplicons were purified using Agencourt AMPure XP beads (Beckman Coulter, UK) using a binding buffer of 2.5 M sodium chloride and 20% PEG-8000, freshly prepared 80% ethanol, and elution buffer (Qiagen, UK). This procedure removes primer dimers and DNA fragments < 200 bp. Samples were pooled into a single library at equimolar concentration and then quantified by using Qubit™ dsDNA HS kit and Qubit 2.0 fluorometer (Thermo Fisher Scientific, UK). DNA quality was checked using a TapeStation automated electrophoresis (Agilent, USA). The final product had 554 bp and an equimolar concentration of 6.3 nM. DNAase free water and an inhouse mock community were used as negative and positive controls respectively. Amplicon libraries were sequenced on the Illumina MiSeq platform (Illumina Cambridge, Ltd, UK), using custom sequencing primers and the MiSeq Reagent Kit v3 (600 cycles, 10% PhiX) to generate paired-end reads.

### Bioinformatics and statistical analyses

Raw 16 S rRNA gene amplicon reads were processed using the Divisive Amplicon Denoising Algorithm 2 (DADA2) (v 1.32.0) [35] in R (v 4.4.2) software to determine amplicon sequence variants (ASVs). Reads quality-filtering and trimming were performed using the *“filterAndTrim”* function (option settings: truncLen = c(250, 240), maxN = 0, maxEE = c(2,2), and truncQ = 2), retaining high-quality bases while ensuring sufficient overlap for forward and reverse read merging. Error rates were modelled using *“learnErrors”* function, followed by read merging (*“mergePairs”*), dereplication (*“derepFastq”*) to identify unique amplicon sequence variants (ASVs), and chimera removal (“*removeBimeraDenovo*”). The final ASV table was constructed using “*makeSequenceTable”*, and taxonomic assignment was performed with *“assignTaxonomy”* against the SILVA version 132 database (genus-level resolution) [36]. Microbial diversity, differential abundance analysis and visualization of microbial diversity was done by Alpha and Beta Diversity analyses using the Phyloseq package v1.48.0. Alpha diversity (within-sample diversity) was assessed using the Shannon index (accounting for species richness and evenness) and Chao1 estimator (taxon richness) using the vegan package v2.6-8 [37]. Comparisons of alpha diversity between groups were performed using the Wilcoxon rank-sum test. Beta diversity (between samples diversity), was analyzed using Bray-Curtis dissimilarity. Ordination was performed using Non-metric Multidimensional Scaling (NMDS) with stress value < 0.17 to visualize sample clustering based on microbial composition. Statistical significance of group differences was assessed using PERMANOVA (Permutational Multivariate Analysis of Variance) via the *“adonis2”* in the vegan package v2.6-8, with 999 permutations to test for robustness [37, 38]. Differential abundance analysis was performed using the DESeq2 package v1.44.0 [39], applying a variance-stabilizing transformation and normalization. Low-abundance taxa present in fewer than 5% of samples were filtered out before analysis. A false discovery rate (FDR)-adjusted p-value of < 0.05 was used to determine significance.

Comparisons of variables between groups was done using Mann-Whitney test or Kruskal-Wallis test when comparing more than two groups for continuous variables; and Chi-square test for categorical variables. Statistical significance for the analyses was set at p-values < 0.05. Additionally, correlations between variables were assessed using Spearman correlation. Statistical programs used for the analyses and visualization include R software v4.4.2 and GraphPad Prism v8.0.2.

## Supplementary Information

Supplementary Material 1.

## Data Availability

16 S rRNA sequencing data generated in this study have been deposited in the NCBI under BioProject accession number PRJNA1294911. Additional data can be obtained from corresponding author upon request.
