# Supplementary material for: Gut microbiome disruption in Tanzanian pulmonary tuberculosis patients: links to treatment, nutritional status, and host immunity
Source: BMC Microbiol. 2026 Mar 26;26:433. doi: 10.1186/s12866-026-04882-3 (PMC13141388; doi:10.1186/s12866-026-04882-3)
Supplement: Supplementary file 1 — Supplementary Material 1. [file 12866_2026_4882_MOESM1_ESM.docx]

# Supplementary Materials

**Supplementary table 1: Sequencing counts** in 49 samples (10 controls, 20 new TB patients and 19 TB patients on treatment) before (Reads in) and after (Reads out) trimming of low-quality reads, denoising (denoisedF/R), merging forward and reverse reads (merged), and after removing chimeras (nonchim).

| **Samples** | **Reads in** | **Reads out** | **denoisedF** | **denoisedR** | **merged** | **nonchim** |
| --- | --- | --- | --- | --- | --- | --- |
| GmCo_001_S1 | 247337 | 187950 | 187471 | 187581 | 185997 | 184632 |
| GmCo_002_S2 | 357012 | 278570 | 278083 | 278114 | 274586 | 263478 |
| GmCo_003_S3 | 256098 | 188572 | 187878 | 188019 | 185894 | 183473 |
| GmCo_004_S4 | 241675 | 183332 | 182168 | 182606 | 178706 | 171147 |
| GmCo_005_S5 | 96904 | 74824 | 74516 | 74528 | 73473 | 72760 |
| GmCo_006_S6 | 252829 | 190408 | 189950 | 189994 | 188687 | 188236 |
| GmCo_007_S7 | 340406 | 248028 | 246922 | 247141 | 242878 | 229213 |
| GmCo_008_S8 | 310578 | 223168 | 222366 | 222478 | 219311 | 212007 |
| GmCo_009_S9 | 314982 | 239253 | 238666 | 238733 | 235283 | 230443 |
| GmCo_010_S10 | 337501 | 251057 | 250129 | 250291 | 247009 | 241340 |
| GmTb_101_S11 | 311844 | 225618 | 224532 | 224536 | 220898 | 207629 |
| GmTb_102_S12 | 137176 | 102524 | 102115 | 102069 | 100209 | 98268 |
| GmTb_103_S13 | 163302 | 121221 | 120359 | 120466 | 118069 | 113515 |
| GmTb_104_S14 | 215211 | 151210 | 150151 | 150416 | 147913 | 144436 |
| GmTb_105_S15 | 144650 | 107683 | 107355 | 107413 | 106564 | 102564 |
| GmTb_106_S16 | 329419 | 248463 | 247450 | 247753 | 244419 | 240356 |
| GmTb_107_S17 | 359095 | 263680 | 262125 | 262398 | 257672 | 252191 |
| GmTb_108_S18 | 359033 | 258825 | 257115 | 257414 | 252991 | 247932 |
| GmTb_109_S19 | 247901 | 179429 | 178934 | 178913 | 177321 | 175917 |
| GmTb_110_S20 | 372348 | 272163 | 270442 | 270435 | 264664 | 256546 |
| GmTb_111_S21 | 229317 | 164347 | 164053 | 164032 | 162839 | 158456 |
| GmTb_112_S22 | 277310 | 205571 | 204862 | 204763 | 201796 | 192873 |
| GmTb_113_S23 | 188056 | 141536 | 141243 | 141244 | 140255 | 135404 |
| GmTb_114_S24 | 233514 | 166441 | 166179 | 166232 | 165585 | 162331 |
| GmTb_115_S25 | 238327 | 179658 | 179172 | 179196 | 177653 | 175762 |
| GmTb_116_S26 | 241441 | 182008 | 181458 | 181561 | 179149 | 177406 |
| GmTb_117_S27 | 251199 | 173255 | 172971 | 172979 | 171917 | 170198 |
| GmTb_118_S28 | 277591 | 194580 | 194250 | 194302 | 193106 | 191113 |
| GmTb_119_S29 | 233329 | 181183 | 180893 | 180865 | 179267 | 178092 |
| GmTb_120_S30 | 235736 | 168323 | 168085 | 168138 | 167270 | 166042 |
| GmTb_201_S31 | 195652 | 142772 | 141879 | 141897 | 137931 | 127405 |
| GmTb_202_S32 | 250622 | 178959 | 178188 | 178107 | 174465 | 158886 |
| GmTb_203_S33 | 197127 | 145418 | 144729 | 144834 | 142812 | 133033 |
| GmTb_204_S34 | 250602 | 164253 | 163037 | 163575 | 160774 | 153107 |
| GmTb_205_S35 | 261564 | 193865 | 193383 | 193450 | 191441 | 188479 |
| GmTb_206_S36 | 228999 | 167563 | 167280 | 167346 | 166489 | 163378 |
| GmTb_207_S37 | 184483 | 137369 | 137087 | 137156 | 135883 | 134103 |
| GmTb_208_S38 | 183574 | 118456 | 118300 | 118350 | 117873 | 117487 |
| GmTb_209_S39 | 190895 | 141913 | 141640 | 141588 | 140400 | 139239 |
| GmTb_210_S40 | 226822 | 161979 | 161653 | 161736 | 160625 | 159222 |
| GmTb_211_S41 | 235194 | 164464 | 164050 | 164125 | 162705 | 158552 |
| GmTb_212_S42 | 225173 | 166795 | 166335 | 166474 | 164355 | 162625 |
| GmTb_213_S43 | 222288 | 156971 | 155933 | 156241 | 153252 | 149023 |
| GmTb_214_S44 | 197506 | 149995 | 149654 | 149770 | 148784 | 139849 |
| GmTb_216_S46 | 261121 | 198956 | 198771 | 198797 | 198207 | 176390 |
| GmTb_217_S47 | 239231 | 179007 | 178713 | 178810 | 177505 | 196393 |
| GmTb_218_S48 | 263969 | 200581 | 200209 | 200323 | 199005 | 120850 |
| GmTb_219_S49 | 181431 | 122223 | 122037 | 122027 | 121528 | 181437 |
| GmTb_220_S50 | 262076 | 190517 | 190272 | 190221 | 189153 | 184632 |
| Total | 12,059,450 | 8,834,936 | 8,805,043 | 8,809,437 | 8,704,568 | 8,467,850 |

**Supplementary table 2: A list of significantly differentially abundant taxa** between TB patient groups and controls.

| **Higher abundance in On_treatment patients vs Controls** | | | | | | |
| --- | --- | --- | --- | --- | --- | --- |
|  | **baseMean** | **log2FoldChange** | **lfcSE** | **stat** | **p-value** | **padj** |
| **Phylum** |  |  |  |  |  |  |
| Fusobacteria | 64.35 | 24.02 | 4.00 | 6.01 | 1.87e-09 | p = 1.87e-08 |
| Tenericutes | 19.43 | 22.35 | 4.00 | 5.59 | 2.28e-08 | p = 1.14e-07 |
| **Genus** |  |  |  |  |  |  |
| Fusobacterium | 25.94 | 23.78 | 4.00 | 5.95 | 2.73e-09 | p = 3.03e-08 |
| Leuconostoc | 26.90 | 23.56 | 4.00 | 5.89 | 3.80e-09 | p = 3.95e-08 |
| Coprobacillus | 7.18 | 21.93 | 4.00 | 5.48 | 4.18e-08 | p = 4.08e-07 |
| Erysipelotrichaceae_UCG-006 | 40.36 | 21.37 | 4.00 | 5.34 | 9.19e-08 | p = 7.27e-07 |
| Parvimonas | 10.84 | 18.32 | 4.01 | 4.57 | 4.80e-06 | p = 3.32e-05 |
| Succinivibrio | 112.65 | 7.68 | 3.01 | 2.56 | 1.06e-02 | p = 4.00e-02 |
| Tyzzerella_4 | 2863.87 | 7.46 | 2.70 | 2.76 | 5.71e-03 | p = 2.43e-02 |
| Solobacterium | 140.09 | 6.52 | 2.49 | 2.61 | 8.94e-03 | p = 3.45e-02 |
| Catenibacterium | 17992.97 | 5.20 | 1.76 | 2.95 | 3.13e-03 | p = 1.53e-02 |
| Holdemanella | 7032.22 | 4.45 | 1.66 | 2.68 | 7.37e-03 | p = 2.91e-02 |
| Lachnoclostridium | 5704.28 | 2.55 | 0.87 | 2.92 | 3.49e-03 | p = 1.66e-02 |
|  |  |  |  |  |  |  |
| **Lower abundance in On_treatment patients vs Controls** | | | | | | |
| **Genus** |  |  |  |  |  |  |
| Turicibacter | 296.46 | -5.94 | 2.40 | -2.48 | 1.33e-02 | p = 4.91e-02 |
|  |  |  |  |  |  |  |
| **Higher abundance in On_treatment patients vs New_patients** | | | | | | |
| **Genus** |  |  |  |  |  |  |
| Prevotellaceae_NK3B31_group | 3.44 | 21.64 | 4.00 | 5.41 | 6.31e-08 | p = 5.52e-07 |
| Megasphaera | 598.44 | 10.75 | 3.97 | 2.71 | 6.71e-03 | p = 2.72e-02 |
| Anaerostipes | 17814.68 | 3.06 | 1.09 | 2.81 | 4.96e-03 | p = 2.17e-02 |
|  |  |  |  |  |  |  |
| **Lower abundance in On_treatment patients vs New_patients** | | | | | | |
| **Phylum** |  |  |  |  |  |  |
| Cyanobacteria | 16.64 | -16.67 | 4.03 | -4.14 | 3.46e-05 | p = 1.15e-04 |
| Euryarchaeota | 41.08 | -11.93 | 4.03 | -2.96 | 3.05e-03 | p = 7.62e-03 |
|  |  |  |  |  |  |  |
| **Genus** |  |  |  |  |  |  |
| Lachnospiraceae_AC2044_group | 892.78 | -29.45 | 2.04 | -14.47 | 2.01e-47 | p = 3.34e-45 |
| Lachnospiraceae_UCG-008 | 45.59 | -26.54 | 2.61 | -10.16 | 3.10e-24 | p = 2.57e-22 |
| Defluviitaleaceae_UCG-011 | 18.64 | -24.67 | 2.76 | -8.95 | 3.71e-19 | p = 2.05e-17 |
| CAG-352 | 590.45 | -30.00 | 3.49 | -8.60 | 8.00e-18 | p = 3.32e-16 |
| Enterobacter | 33.88 | -25.61 | 3.06 | -8.37 | 5.74e-17 | p = 1.91e-15 |
| Sellimonas | 233.15 | -26.32 | 3.29 | -8.01 | 1.19e-15 | p = 3.31e-14 |
| Lactonifactor | 38.36 | -25.22 | 3.68 | -6.85 | 7.44e-12 | p = 1.76e-10 |
| Adlercreutzia | 22.49 | -26.16 | 3.97 | -6.58 | 4.57e-11 | p = 9.49e-10 |
| Ruminococcaceae_UCG-004 | 101.27 | -24.78 | 3.97 | -6.24 | 4.47e-10 | p = 6.19e-09 |
| Ruminococcaceae_UCG-009 | 224.70 | -24.83 | 3.89 | -6.39 | 1.71e-10 | p = 2.84e-09 |
| Rikenellaceae_RC9_gut_group | 103.96 | -24.91 | 3.97 | -6.27 | 3.62e-10 | p = 5.46e-09 |
| Eubacterium | 46.42 | -24.45 | 3.97 | -6.15 | 7.62e-10 | p = 9.73e-09 |
| Ruminococcaceae_UCG-014 | 213.47 | -25.77 | 3.97 | -6.48 | 8.88e-11 | p = 1.64e-09 |
| Weissella | 30.77 | -23.82 | 3.97 | -5.99 | 2.05e-09 | p = 2.44e-08 |
| Eisenbergiella | 17.11 | -21.47 | 3.98 | -5.39 | 6.87e-08 | p = 5.70e-07 |
| Butyricimonas | 10.36 | -21.51 | 3.94 | -5.46 | 4.65e-08 | p = 4.29e-07 |
| DNF00809 | 12.00 | -18.76 | 4.03 | -4.66 | 3.17e-06 | p = 2.29e-05 |
| Anaerotruncus | 16.64 | -19.03 | 4.01 | -4.75 | 2.02e-06 | p = 1.52e-05 |
| Ruminococcaceae_UCG-002 | 11611.29 | -3.87 | 1.19 | -3.26 | 1.12e-03 | p = 6.42e-03 |
| UBA1819 | 117.50 | -5.74 | 1.68 | -3.41 | 6.39e-04 | p = 4.20e-03 |
| Oscillibacter | 525.57 | -6.53 | 1.92 | -3.41 | 6.57e-04 | p = 4.20e-03 |
| Lachnospiraceae_UCG-001 | 42.24 | -9.81 | 2.99 | -3.28 | 1.03e-03 | p = 6.09e-03 |
| Megamonas | 133.16 | -12.57 | 4.03 | -3.12 | 1.79e-03 | p = 9.91e-03 |
| Negativibacillus | 72.59 | -13.54 | 4.03 | -3.36 | 7.66e-04 | p = 4.71e-03 |
| Ruminococcaceae_NK4A214_group | 1880.20 | -4.42 | 1.53 | -2.89 | 3.91e-03 | p = 1.80e-02 |
| Candidatus_Soleaferrea | 63.40 | -4.53 | 1.60 | -2.84 | 4.56e-03 | p = 2.04e-02 |
| Clostridium_sensu_stricto_1 | 1461.31 | -4.86 | 1.62 | -3.01 | 2.64e-03 | p = 1.33e-02 |
| Family_XIII_AD3011_group | 2672.89 | -4.93 | 1.61 | -3.07 | 2.15e-03 | p = 1.15e-02 |
| GCA-900066225 | 22.11 | -5.63 | 2.06 | -2.73 | 6.37e-03 | p = 2.65e-02 |
| Ruminococcaceae_UCG-010 | 2196.96 | -6.30 | 2.10 | -3.01 | 2.65e-03 | p = 1.33e-02 |

baseMean is the average normalized count of the taxon across all samples; log2foldchange is the log2-transformed fold change in abundance between two groups (e.g., treatment vs. control), with positive and negative values reflecting higher or lower abundance in the reference group respectively; IfcSE is the standard error of the log2 fold change; stat is the Wald test statistic (ratio of log2FoldChange / lfcSE) used to compute the p-value; and padj is the adjusted p-value using Benjamini-Hochberg FDR correction to account for multiple testing.

**Supplementary figures**


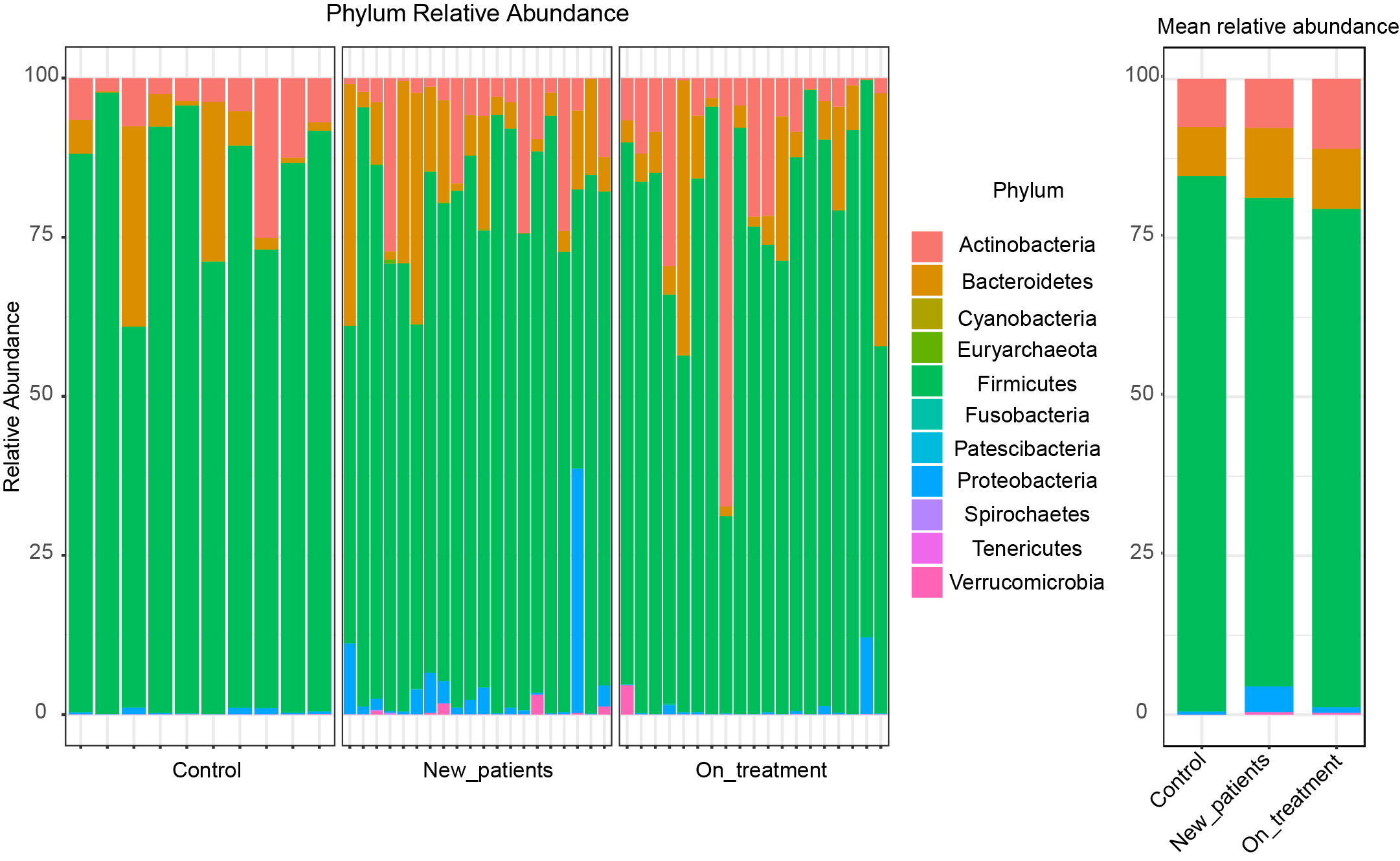


**Supplementary figure 1: Relative abundance of phyla across TB patients and Controls**. The left panel shows relative abundance in individual samples while the right panel shows the average relative abundance in each group.


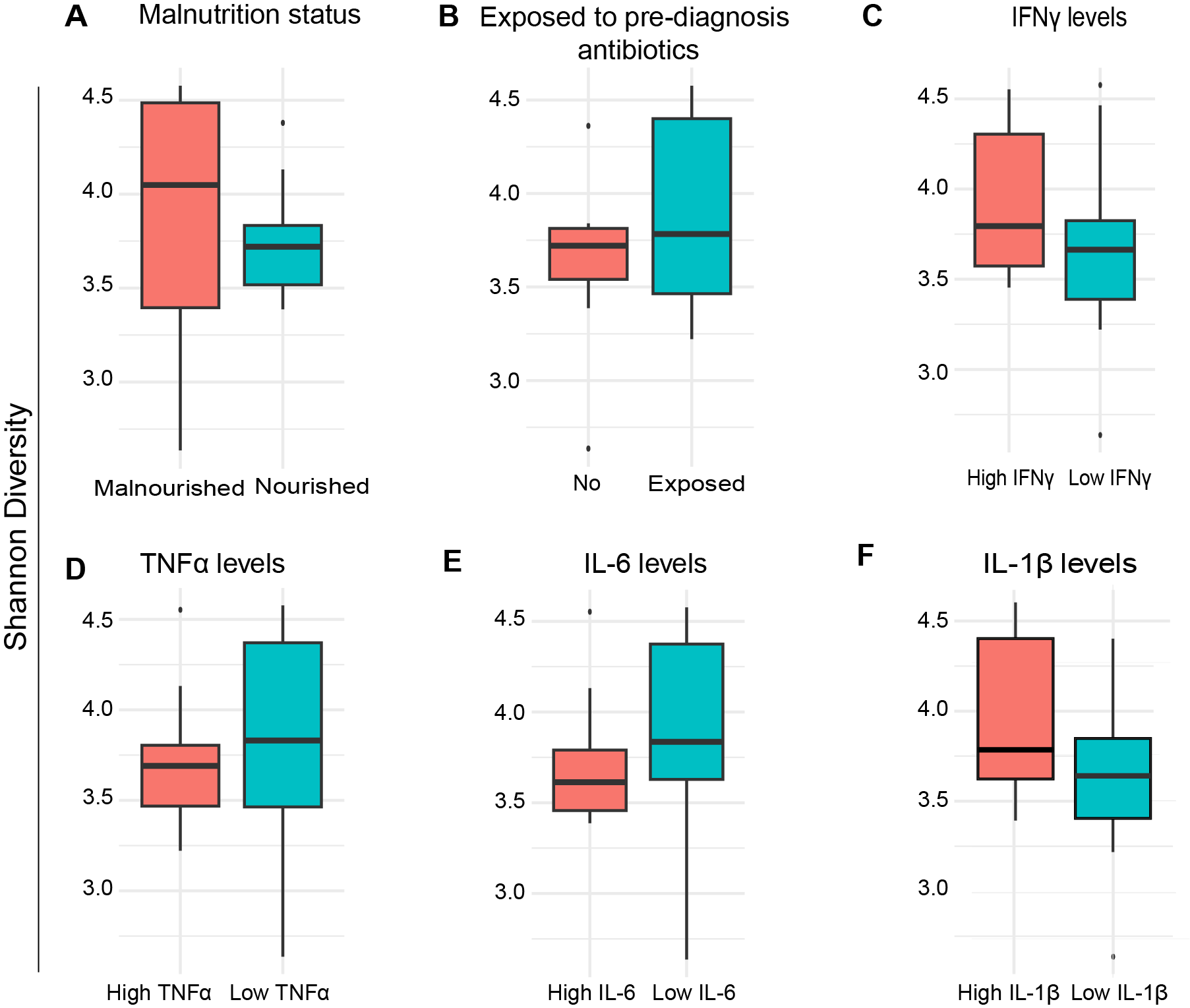


**Supplementary figure 2. Alpha diversity across newly diagnosed** **TB patients with different clinical characteristics. (A-F)** Box plots showing Shannon diversity indices across newly diagnosed TB patients with different characteristics including with and without malnutrition (A), with and without exposure to empirical antibiotics (B), and with high and low cytokine levels (C-F). The central line represents the median, the box indicates the interquartile range (IQR), and whiskers extend to 1.5 times the IQR. Outliers are shown as individual points. Differences in alpha diversity reflect variation in microbial richness and evenness between groups.
